# Safety and Efficacy of Vadadustat for the Treatment of CKD-Related Anemia within and outside the United States

**DOI:** 10.1681/ASN.0000000708

**Published:** 2025-05-13

**Authors:** Glenn M. Chertow, Kai-Uwe Eckardt, Mark J. Sarnak, Wolfgang C. Winkelmayer, Rajiv Agarwal, Todd Minga, Wenli Luo, Steven K. Burke

**Affiliations:** 1 Departments of Medicine, Epidemiology and Population Health, and Health Policy, Stanford University School of Medicine, Stanford, California; ^2^Department of Nephrology and Medical Intensive Care, Charité–Universitätsmedizin Berlin, Berlin, Germany; 3 Department of Medicine, Tufts Medical Center, Boston, Massachusetts; 4 Department of Medicine, Baylor College of Medicine, Houston, Texas; 5 Department of Medicine, Richard L. Roudebush Veterans Administration Medical Center, Indiana University School of Medicine, Indianapolis, Indiana; 6 Akebia Therapeutics, Inc., Cambridge, Massachusetts

**Keywords:** anemia, cardiovascular events, CKD, clinical trial, ESKD

## Abstract

**Key Points:**

- Among patients with dialysis-dependent CKD, safety and efficacy of vadadustat and darbepoetin alfa in the United States and outside the United States were similar.
- Among US patients with non–dialysis-dependent CKD, safety outcomes were similar; the relative risk for major adverse cardiovascular event with vadadustat was higher outside the United States.
- Region-specific analyses reflect differences in trial patient characteristics, hemoglobin targets, and access to health care services.

**Background:**

Vadadustat's global clinical program was comprised of four noninferiority trials comparing vadadustat and darbepoetin alfa for CKD-related anemia: two in dialysis-dependent CKD (DD-CKD) and two in non–dialysis-dependent CKD (NDD-CKD). Although vadadustat met prespecified noninferiority criteria for hematologic efficacy globally, it did not meet noninferiority criteria for cardiovascular safety in the non–dialysis-dependent CKD trials. The trials considered regional differences in treatment practices, including hemoglobin targets within (10–11 g/dl) and outside the United States (10–12 g/dl).

**Methods:**

To examine region-specific outcomes, we performed prespecified analyses for US and non-US patient subgroups from the vadadustat global program. The primary safety end point was first occurrence of major adverse cardiovascular event (MACE; death from any cause or nonfatal myocardial infarction or stroke). The primary efficacy end point was change in hemoglobin from baseline to average values during weeks 24–36.

**Results:**

Four thousand eighty-four/7399 (55%) randomized patients were enrolled in the United States. In pooled analyses of all US patients, MACE risk was similar among vadadustat-treated and darbepoetin alfa–treated patients (hazard ratio [HR], 1.03; 95% confidence interval [CI], 0.90 to 1.17). HRs were similar for US patients with DD-CKD (HR, 1.00; 95% CI, 0.84 to 1.18) and NDD-CKD (HR, 1.06; 95% CI, 0.87 to 1.29). In pooled analyses of non-US patients, MACE risk was numerically higher among vadadustat-treated patients (HR, 1.12; 95% CI, 0.94 to 1.33); the higher risk was primarily attributed to the NDD-CKD subgroup (HR, 1.29; 95% CI, 1.03 to 1.60). In the non-US DD-CKD subgroup, MACE risk was similar among vadadustat-treated and darbepoetin alfa–treated patients (HR, 0.88; 95% CI, 0.67 to 1.17). Changes in hemoglobin were similar among treatment groups in all regions, as were rates of treatment-emergent and serious adverse events.

**Conclusions:**

In patients with DD-CKD, safety (vis-à-vis MACE) and efficacy (vis-à-vis change in hemoglobin) of vadadustat and darbepoetin alfa were similar when stratified by region (US versus non-US). In US patients with NDD-CKD, safety and efficacy of vadadustat and darbepoetin alfa were similar.

**Clinical Trial registry name and registration number:**

NCT02865850, NCT02892149, NCT02648347, NCT02680574.

## Visual Abstract

**Figure:**
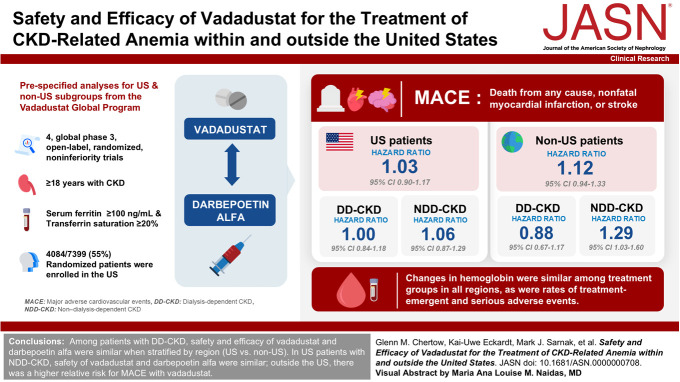

## Introduction

Anemia is a common complication of CKD that typically worsens with disease progression.^1–3^ Conventional treatments for CKD-related anemia include iron supplements, erythropoiesis-stimulating agents (ESAs), and red blood cell (RBC) transfusion.^4,5^ Parenteral administration of ESAs often requires dedicated visits to a health care facility or infusion center.^4,6,7^ Alternative treatment options for CKD-related anemia now include oral hypoxia-inducible factor prolyl hydroxylase inhibitors (HIF-PHIs).^8,9^

HIF-PHIs stabilize hypoxia-inducible factor, a transcription factor that regulates hypoxia-sensitive genes, enhancing endogenous erythropoietin production and iron availability.^1,10,11^ Vadadustat is an oral HIF-PHI developed for the treatment of CKD-related anemia.^12–14^ Vadadustat is approved for the treatment of CKD-related anemia in Japan and for patients with dialysis-dependent CKD (DD-CKD) in the United States, Europe, and Australia.^9,15–17^

The vadadustat global phase 3 clinical program included two trials in patients with DD-CKD (INNO_2_VATE) and two trials in patients with non–dialysis-dependent CKD (NDD-CKD; PRO_2_TECT).^12,13^ In the primary analysis of the combined INNO_2_VATE trials, vadadustat was noninferior to darbepoetin alfa for cardiovascular safety (major adverse cardiovascular event [MACE]: all-cause mortality, nonfatal myocardial infarction [MI], and nonfatal stroke), with a hazard ratio (HR) of 0.96 (95% confidence interval [CI], 0.83 to 1.11), not exceeding the prespecified upper bound of the noninferiority margin of 1.25.^12^ In the primary analysis of the combined PRO_2_TECT trials, vadadustat exceeded the upper bound of noninferiority margin of 1.25 (HR, 1.17; 95% CI, 1.01 to 1.36) and thus did not meet the noninferiority criteria for cardiovascular safety.^13^ Vadadustat was noninferior to darbepoetin alfa for hematologic efficacy across all INNO_2_VATE and PRO_2_TECT trials.^12,13^

The phase 3 global program was designed to align with regional differences in treatment practice; specifically, hemoglobin targets differed on the basis of existing regional ESA regulatory labeling. The hemoglobin target was 10–11 g/dl in the United States and 10–12 g/dl outside of the United States.^12,13^ Given the variation in hemoglobin targets and treatment patterns among geographic regions, we conducted prespecified subgroup analyses of US and non-US patients to look more closely at region-specific safety and efficacy outcomes for this patient population.

Here, we describe the safety and efficacy of vadadustat compared with darbepoetin alfa in US and non-US patients enrolled in the INNO_2_VATE and PRO_2_TECT trials.

## Methods

### Trial Design

We conducted four global phase 3, open-label, randomized, noninferiority trials that compared the safety and efficacy of vadadustat with darbepoetin alfa in adult patients with CKD-related anemia. The rationale, design, methods, and primary results of the trials have been previously reported.^12,13^

The INNO_2_VATE program included two randomized, active ESA (darbepoetin alfa), controlled, open-label, noninferiority clinical trials in patients with DD-CKD. One trial was dedicated to patients with incident (<16 weeks) DD-CKD (NCT02865850, *n*=369); another was dedicated to patients with prevalent (≥12 weeks) DD-CKD (NCT02892149, *n*=3554).^12^

The PRO_2_TECT program included two randomized, active ESA (darbepoetin alfa), controlled, open-label, noninferiority clinical trials in patients with NDD-CKD. One trial included patients who had not recently been treated with an ESA (NCT02648347, *n*=1751); the other trial included patients who were previously treated with an ESA (NCT02680574, *n*=1725).^13^

All trials were performed in compliance with the International Conference on Harmonisation, in accordance with Good Clinical Practice guidelines and applicable local regulatory requirements and laws, and in line with the principles of the Declaration of Helsinki. Institutional review board approval was obtained at each participating site. All patients provided written informed consent before enrollment.

### Trial Population

In all four trials, eligible patients were aged 18 years or older, had been diagnosed with CKD, and had serum ferritin concentrations of ≥100 ng/ml and transferrin saturation of ≥20%. In the INNO_2_VATE trials, eligible patients were adults with DD-CKD, with no RBC transfusions within 8 weeks before randomization. Patients in the incident DD-CKD trial were required to have a hemoglobin concentration of 8–11 g/dl. The prevalent DD-CKD trial enrolled patients in the United States who had hemoglobin concentrations of 8–11 g/dl and patients in other countries who had hemoglobin concentrations of 9–12 g/dl.^12^ The PRO_2_TECT trials enrolled adult patients with NDD-CKD and an eGFR ≤60 ml/min per 1.73 m^2^ of body surface area. ESA-untreated patients were required to have had hemoglobin concentrations <10 g/dl with no ESA use within 8 weeks before enrollment. Patients treated with an ESA were eligible if they had received maintenance ESA therapy with at least one dose within 6 weeks before screening, with a hemoglobin concentration of 8–11 g/dl in the United States and 9–12 g/dl in other countries.^12,13^

### Study Procedures

Eligible patients were randomized 1:1 to receive vadadustat or darbepoetin alfa, stratified by geographic region (United States/Europe/other regions), New York Heart Association (NYHA) congestive heart failure class (0/1 versus 2/3), and hemoglobin concentration (<9.5 or ≥9.5 g/dl) at entry. The trials had four defined periods: (*1*) correction or conversion period (weeks 0–23); (*2*) maintenance period (weeks 24–52), which included the primary (weeks 24–36) and secondary (weeks 40–52) efficacy periods; (*3*) long-term safety period (weeks 53 to end of treatment [182 weeks]); and (*4*) 4-week safety follow-up period after the end of treatment (Supplemental Figure 1).

The vadadustat starting dose was 300 mg orally once daily (two 150 mg tablets), with doses of 150, 300, 450, and 600 mg available for adjustment to a maximum daily dose of 600 mg. Darbepoetin alfa was administered intravenously for patients on maintenance hemodialysis and subcutaneously for patients receiving peritoneal dialysis by staff at the site facility or by the participant at home according to the investigator's determination and local practice. The initial dose of darbepoetin alfa was based on dose before randomization for those patients already receiving darbepoetin alfa or on the local product label for patients not receiving darbepoetin alfa before randomization but switching from a different ESA. Doses of vadadustat and darbepoetin alfa were titrated according to protocol-specified dose adjustment guidelines to maintain target hemoglobin concentrations (10–11 g/dl for patients in the United States and 10–12 g/dl in other countries). Iron supplementation was encouraged to maintain serum ferritin concentrations ≥100 ng/ml or transferrin saturation ≥20%. During the first year of treatment, we measured hemoglobin concentrations every 2 weeks for weeks 0–12 and every 4 weeks for weeks 12–52 and adjusted drug doses accordingly. Site investigators aimed to maintain hemoglobin concentrations within the geography-specific target range. Trial medications were adjusted according to the investigator's clinical discretion, incorporating protocol-based guidance and considering the patient's clinical condition and the trajectory of hemoglobin concentrations.

Starting at 6 weeks, patients in both treatment groups could receive either an ESA or RBC transfusion as “rescue therapy” if they experienced worsening symptoms of anemia with hemoglobin concentrations <9.5 g/dl. In the vadadustat group, rescue was defined as an RBC transfusion or ESA therapy. In the darbepoetin alfa group, rescue was defined as an RBC transfusion, a dose increase of at least double the previous darbepoetin alfa dose, or another ESA per the standard of care. In the event of an acute or severe loss of blood, an RBC transfusion was administered as clinically indicated. In cases where there was worsening of anemia or moderate to severe symptoms of anemia, RBC transfusions were permitted. Trial drugs were continued during the transfusion period.

### End Points

In the INNO_2_VATE and PRO_2_TECT programs, the primary safety end point was the first occurrence of an adjudicated MACE (all-cause mortality, nonfatal MI or stroke) using a time-to-event analysis.^12,13^ Secondary safety assessments included all-cause mortality, first occurrence of expanded MACE (MACE plus hospitalization for heart failure or thromboembolic events, excluding vascular access failure), first occurrence of cardiovascular (“classic”) MACE (cardiovascular mortality, nonfatal MI, or nonfatal stroke), and cardiovascular mortality.^12,13^

The primary efficacy end point across the clinical programs was mean change in hemoglobin concentration from baseline to the primary evaluation period (weeks 24–36); the key secondary end point was mean change in hemoglobin concentration from baseline to the secondary evaluation period (weeks 40–52).^12,13^

In addition, we assessed the number of patients who had an average hemoglobin concentration within the US- and non–US-specific target ranges during the primary and secondary evaluation periods as well as incidence of adverse events (AEs), including treatment-emergent AEs and serious AEs (SAEs), for all trials.

We prespecified the use of pooled data from both DD-CKD and both NDD-CKD trials for the US and non-US as well as all four trials for safety analyses, specifically AEs and SAEs. *Post hoc* analyses were conducted from the pooled data for risk of MACE. Efficacy analyses were conducted separately by individual trial.

### Statistical Analyses

Analyses were conducted for the prespecified US and non-US subgroups from the INNO_2_VATE and PRO_2_TECT trials. A list of countries in which the INNO_2_VATE and PRO_2_TECT trials were conducted is presented in Supplemental Table 1. We analyzed time to first MACE on the basis of a stratified Cox regression model with study as a stratification factor. The model included covariates of baseline hemoglobin, randomization strata of NYHA (0 or 1; 2 or 3), sex, age (younger than 65 years; 65 years or older), race (White or non-White), preexisting cardiovascular disease (yes/no), and diabetes mellitus (yes/no). Since randomization was imbalanced by age stratum (<65, ≥65), we conducted a series of *post hoc* companion analyses where we adjusted for age as a continuous variable. We calculated the HR (vadadustat/darbepoetin alfa) and its 95% CI from model parameter estimates and standard errors, respectively. As we did for the primary analysis of the full trials, we determined noninferiority on MACE if the upper limit of the 95% CI was <1.25. For the primary efficacy end point, we used a two-sided 95% CI with a lower bound noninferiority margin of −0.75 g/dl (vadadustat minus darbepoetin alfa) to evaluate the difference between treatment groups, as previously described.^14^

In efficacy analyses, we used multiple imputation with analysis of covariance, comparing groups based on treatment, baseline hemoglobin, and stratification factors (region, NYHA congestive heart failure class). We addressed missing data through multiple imputation using a fully conditional specification approach. Proportion differences were calculated using the Mantel–Haenszel method by three randomization stratification factors. Within any stratum, if there were no patients in any treatment group or no responders in both treatment groups, unstratified Mantel–Haenszel method was used for analysis.

## Results

The first patient enrolled in the INNO_2_VATE program on July 18, 2016, and the last patient visit was on January 31, 2020. In the PRO_2_TECT program, the first patient enrolled on December 17, 2015, and the last patient visit was on June 18, 2020. Both programs ended when there were at least 631 MACE accrued in each program. The 7373 randomized and treated patients from the global INNO_2_VATE and PRO_2_TECT programs consisted of 4084 (55%) US patients and 3289 (45%) non-US patients (Supplemental Figure 2).

### Demographics and Baseline Characteristics

Overall demographics and baseline characteristics in the pooled US and non-US populations were generally balanced between the treatment groups within the DD-CKD and NDD-CKD programs (Table 1). In all trials, between regions, there were several key differences; there was a higher percentage of Black patients, higher weight and body mass index, higher prior ESA dose, substantially more patients with diabetes, and more patients with cardiovascular disease in the US compared with non-US populations (Table 1). In the INNO_2_VATE trials, median serum ferritin concentrations in US patients were nearly double those of non-US patients (891 ng/ml [interquartile range (IQR), 648 ng/ml] and 868 ng/ml [IQR, 666 ng/ml] in the vadadustat and darbepoetin groups in US patients versus 424 ng/ml [IQR, 538 ng/ml] and 459 ng/ml [IQR, 539 ng/ml], respectively, in non-US patients), and the proportion of patients receiving peritoneal dialysis was lower (6% and 8%, respectively, in US patients versus 10% and 9%, respectively, in non-US patients). In the PRO_2_TECT trials, the proportion of patients with stage G5 CKD (eGFR <15 ml/min per 1.73 m^2^) was substantially higher in non-US patients (38% and 34% in the vadadustat and darbepoetin groups versus 25% and 27%, respectively, in US patients), as was the proportion of patients with eGFR <10 ml/min per 1.73 m^2^ (16% and 14% in the vadadustat and darbepoetin groups versus 7% and 7%, respectively, in US patients; Table 1).

**Table 1:** Demographics and baseline characteristics of US and non-US patients in the INNO_2_VATE (dialysis-dependent CKD) and PRO_2_TECT (non–dialysis-dependent CKD) safety populations

Characteristic	US				Non-US			
	INNO_2_VATE DD-CKD Trials		PRO_2_TECT NDD-CKD Trials		INNO_2_VATE DD-CKD Trials		PRO_2_TECT NDD-CKD Trials	
	Vadadustat (*n*=1180)	Darbepoetin Alfa (*n*=1181)	Vadadustat (*n*=861)	Darbepoetin Alfa (*n*=862)	Vadadustat (*n*=767)	Darbepoetin Alfa (*n*=774)	Vadadustat (*n*=878)	Darbepoetin Alfa (*n*=870)
Mean age, yr, (SD)	59 (14)	59 (14)	69 (13)	68 (13)	56 (14)	57 (14)	64 (14)	64 (14)
**Age category, yr, *n* (%)**								
<65	755 (64)	754 (64)	303 (35)	309 (36)	525 (68)	538 (70)	406 (46)	401 (46)
≥65	425 (36)	427 (36)	558 (65)	553 (64)	242 (32)	236 (31)	472 (54)	469 (54)
Sex, male, *n* (%)	651 (55)	647 (55)	415 (48)	358 (42)	438 (57)	463 (60)	382 (44)	380 (44)
**Racial or ethnic group, *n* (%)**								
Asian	28 (2)	43 (4)	39 (5)	25 (3)	60 (8)	63 (8)	71 (8)	67 (8)
Black or African American	411 (35)	423 (36)	236 (27)	263 (31)	59 (8)	55 (7)	44 (5)	39 (5)
Other^a^	80 (7)	89 (8)	27 (3)	30 (3)	54 (7)	51 (7)	145 (17)	136 (16)
White	661 (56)	626 (53)	559 (65)	544 (63)	594 (77)	605 (78)	618 (70)	628 (72)
**Hispanic ethnic group, *n* (%)**								
Hispanic/Latino	534 (45)	515 (44)	275 (32)	266 (31)	216 (28)	220 (28)	286 (33)	299 (34)
Not Hispanic/Latino	635 (54)	655 (56)	583 (68)	591 (69)	505 (66)	498 (64)	566 (65)	551 (63)
Weight, kg, mean (SD)	84 (23)	84 (23)	85 (24)	86 (24)	74 (18)	73 (17)	75 (17)	75 (17)
BMI, kg/m^2^, mean (SD)	30.0 (7.7)	29.9 (7.6)	30.8 (7.9)	31.4 (8.0)	26.2 (5.4)	26.2 (5.4)	28.0 (6.0)	28.1 (6.1)
**NYHA FC, *n* (%)**								
0 or 1	1027 (87)	1028 (87)	774 (90)	774 (90)	671 (88)	672 (87)	721 (82)	716 (82)
2 or 3	153 (13)	153 (13)	87 (10)	88 (10)	96 (13)	102 (13)	157 (18)	154 (18)
**Type of ESA, *n***	1081	1080	317	324	677	686	515	518
Epoetin, *n* (%)	497 (46)	477 (44)	227 (72)	238 (74)	468 (69)	486 (71)	283 (55)	285 (55)
Darbepoetin alfa, *n* (%)	331 (31)	363 (34)	90 (28)	86 (27)	151 (22)	157 (23)	171 (33)	187 (36)
Methoxy polyethylene glycol-epoetin *β*	253 (23)	240 (22)	0	0	58 (9)	43 (6)	61 (12)	46 (9)
**Baseline ESA dose (U/kg per week), *n* (%)**	1069	1072	315	319	666	679	508	516
Mean (SD)	124 (124)	116 (120)	149 (192)	131 (141)	105 (79)	105 (88)	77 (91)	89 (258)
≤90 U/kg per week	563 (53)	588 (55)	174 (55)	164 (51)	350 (53)	375 (55)	376 (74)	394 (76)
>90 and <300 U/kg per week	421 (39)	404 (38)	102 (3)	127 (40)	299 (45)	287 (42)	119 (23)	111 (22)
≥300 U/kg per week	85 (8)	80 (8)	39 (12)	28 (9)	17 (3)	17 (3)	13 (3)	11 (2)
**Comorbidities, *n* (%)**								
Diabetes	820 (70)	818 (69)	612 (71)	616 (72)	250 (33)	270 (35)	486 (55)	499 (57)
Cardiovascular disease^b^	630 (53)	678 (57)	423 (49)	434 (50)	304 (40)	323 (42)	358 (41)	379 (44)
**Etiology of CKD, *n* (%)^c^**								
Diabetes	669 (57)	665 (56)	544 (63)	532 (62)	201 (26)	231 (30)	407 (46)	412 (47)
Hypertension	707 (60)	717 (61)	557 (65)	547 (64)	259 (34)	272 (35)	417 (48)	403 (46)
Autoimmune/glomerulonephritis/vasculitis	45 (34)	58 (5)	34 (4)	39 (5)	153 (20)	155 (20)	89 (10)	78 (9)
Cystic/hereditary/congenital disease	19 (2)	17 (1)	12 (1)	15 (2)	56 (7)	54 (7)	34 (4)	25 (3)
Other^d^	88 (7)	101 (9)	74 (9)	75 (9)	224 (29)	217 (28)	208 (24)	223 (26)
**Years since CKD diagnosis, *n***	1124	1123	849	858	762	767	873	865
Median (Q1–Q3)	4.0 (1.8–7.6)	3.9 (1.8–7.6)	3.3 (1.5–6.6)	3.8 (1.5–7.3)	6.0 (2.6–11.0)	6.3 (2.8–11.3)	3.6 (1.4–7.7)	3.1 (1.0–6.4)
**eGFR, ml/min per 1.73 m** ^**2**^ **, *n* (%)**								
<10	NA	NA	56 (7)	60 (7)	NA	NA	143 (16)	125 (14)
≥10	NA	NA	805 (93)	802 (93)	NA	NA	735 (84)	745 (86)
<15	NA	NA	212 (25)	235 (27)	NA	NA	333 (38)	299 (34)
≥15	NA	NA	649 (75)	627 (73)	NA	NA	545 (62)	571 (66)
**Type of dialysis, *n* (%)**	1179	1180	NA	NA	766	774	NA	NA
Hemodialysis	1106 (94)	1090 (92)	NA	NA	687 (90)	707 (91)	NA	NA
Peritoneal	73 (6)	90 (8)	NA	NA	79 (10)	67 (9)	NA	NA
**Dialysis access type, *n* (%)**								
Arteriovenous fistula	797 (68)	784 (66)	NA	NA	568 (74)	560 (72)	NA	NA
Arteriovenous graft	145 (12)	147 (12)	NA	NA	15 (2)	33 (4)	NA	NA
Temporary catheter	21 (2)	25 (2)	NA	NA	22 (3)	21 (3)	NA	NA
Tunneled dialysis catheter	145 (12)	132 (11)	NA	NA	89 (12)	94 (12)	NA	NA
Other^e^	72 (6)	93 (8)	NA	NA	73 (10)	66 (9)	NA	NA
**Years since maintenance dialysis initiation, *n***	1179	1179	NA	NA	765	774	NA	NA
Median (Q1–Q3)	2.3 (1.0–4.9)	2.4 (0.9–4.8)	NA	NA	2.2 (0.8–5.4)	2.0 (0.7–5.0)	NA	NA
Incident dialysis, *n* (%)	121 (10)	133 (11)	NA	NA	101 (13)	117 (15)	NA	NA
Hb, g/dl, mean (SD)	10.0 (0.8)	10.0 (0.8)	9.4 (0.8)	9.4 (0.8)	10.5 (1.0)	10.4 (1.0)	10.1 (1.2)	10.1 (1.2)
Ferritin, ng/ml, median (Q1–Q3)	891 (569–1217)	868 (552–1218)	302 (167–484)	292 (180–498)	424 (255–793)	459 (277–816)	269 (170–451)	259 (168–420)
TSAT (%), mean (SD)	38 (13)	38 (13)	31 (9)	32 (10)	36 (14)	36 (13)	32 (10)	32 (10)

BMI, body mass index; DD-CKD, dialysis-dependent CKD; ESA, erythropoiesis-stimulating agent; Hb, hemoglobin; NA, not applicable; NDD-CKD, non–dialysis-dependent CKD; NYHA FC, New York Heart Association Functional Class; TSAT, transferrin saturation; US, United States.

a Includes American Indian or Alaska Native (*n*=17, 29, 2, 2, 3, 1, 52, 47, respectively, across columns), Native Hawaiian or other Pacific Islander (*n*=12, 6, 7, 5, 1, 0, 2, 1, respectively, across columns), multiple (*n*=4, 3, 1, 7, 5, 2, 6, 5, respectively, across columns), any other (*n*=11, 15, 9, 8, 30, 31, 74, 72, respectively, across columns), or not reported (*n*=36, 36, 8, 8, 15, 17, 11, 11, respectively, across columns).

b Includes coronary artery disease, myocardial infarction, stroke, and heart failure.

c A patient could have more than one CKD etiology.

d Includes interstitial nephritis/pyelonephritis, neoplasms/tumors, or any other.

e Includes missing or any other.

### Time to First MACE

**Figure 1:**
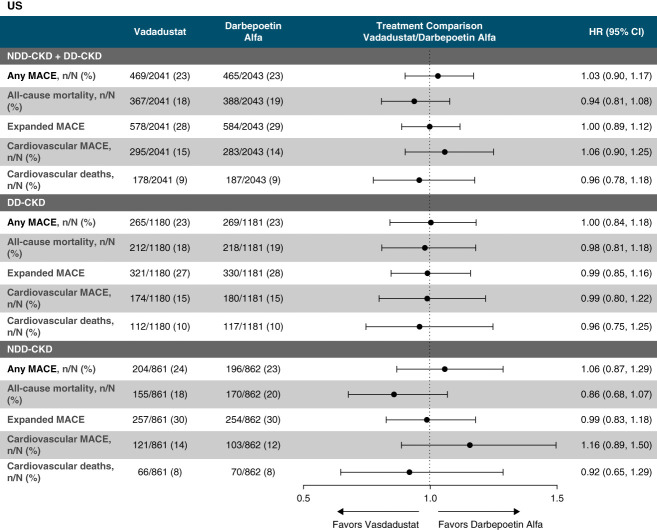

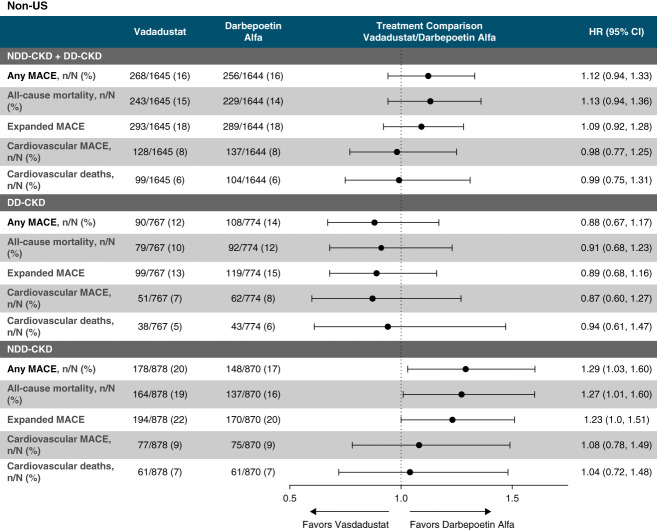
**Risk of MACE and MACE-related outcomes in US and non-US patients from the INNO**_**2**_**VATE (DD-CKD) and PRO**_**2**_**TECT (NDD-CKD) trials.** CI, confidence interval; DD-CKD, dialysis-dependent CKD; HR, hazard ratio; MACE, major adverse cardiovascular event; NDD-CKD, non–dialysis-dependent CKD; US, United States.

**Figure 2:**
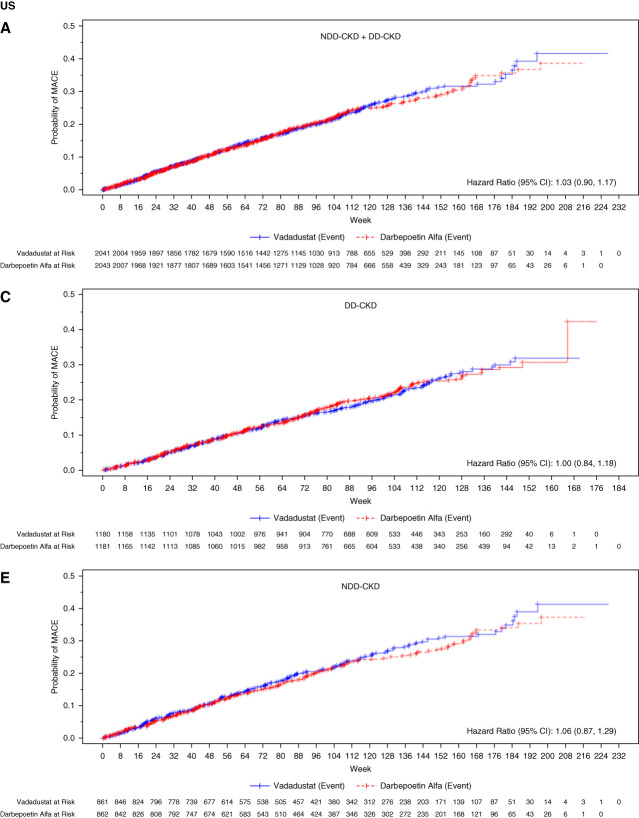

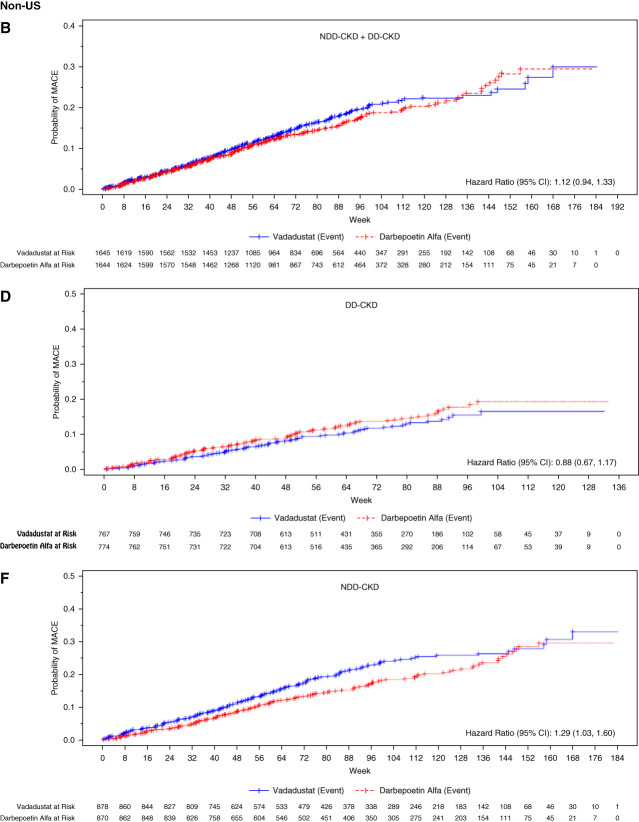
**Risk of MACE in US and non-US patients from the INNO**_**2**_**VATE (DD-CKD) and PRO**_**2**_**TECT (NDD-CKD) trials.** (A) MACE in US patients with NDD-CKD or DD-CKD. (B) MACE in non-US patients with NDD-CKD or DD-CKD. (C) MACE in US patients with DD-CKD. (D) MACE in non-US patients with DD-CKD. (E) MACE in US patients with NDD-CKD. (F) MACE in non-US patients with NDD-CKD.

Based on pooled analyses of the US population of the two programs (four trials), the risk of MACE was similar in vadadustat-treated (469/2041 [23.0%]) and darbepoetin alfa–treated (465/2043 [22.8%]) patients (HR, 1.03; 95% CI, 0.90 to 1.17; Figures 1 and 2A and Table 2). The risk of MACE was also similar when evaluating US patients with DD-CKD or NDD-CKD separately; a first MACE occurred in 265/1180 (23%) US patients randomized to vadadustat and in 269/1181 (23%) US patients randomized to darbepoetin alfa in the INNO_2_VATE trials (HR, 1.00; 95% CI, 0.84 to 1.18; Figures 1 and 2C and Table 2). In the PRO_2_TECT trials, first MACE occurred in 204/861 (24%) US patients randomized to vadadustat and 196/862 (23%) patients randomized to darbepoetin alfa (HR, 1.06; 95% CI, 0.87 to 1.29; Figures 1 and 2E and Table 2). Supplemental Figure 3 shows HRs and 95% CI for safety end points for the pooled, DD-CKD, and NDD-CKD populations when adjusting for age as a continuous variable in the United States.

**Table 2:** Risk of major adverse cardiovascular event in US and non-US patients from the INNO_2_VATE (dialysis-dependent CKD) and PRO_2_TECT (non–dialysis-dependent CKD) trials

MACE	Vadadustat	Darbepoetin Alfa	HR (95% CI)
**US**			
NDD+DD-CKD			
*Any MACE, n/N (%)*	469/2041 (23)	465/2043 (23)	1.03 (0.90 to 1.17)
DD-CKD			
*Any MACE, n/N (%)*	265/1180 (23)	269/1181 (23)	1.00 (0.84 to 1.18)
NDD			
*Any MACE, n/N (%)*	204/861 (24)	196/862 (23)	1.06 (0.87 to 1.29)
**Non-US**			
NDD+DD-CKD			
*Any MACE, n/N (%)*	268/1645 (16)	256/1644 (16)	1.12 (0.94 to 1.33)
DD-CKD			
*Any MACE, n/N (%)*	90/767 (12)	108/774 (14)	0.88 (0.67 to 1.17)
NDD			
*Any MACE, n/N (%)*	178/878 (20)	148/870 (17)	1.29 (1.03 to 1.60)
**Overall**			
NDD+DD-CKD			
*Any MACE, n/N (%)*	737/3686 (20.0)	721/3687 (19.6)	1.06 (0.96 to 1.18)
DD-CKD			
*Any MACE, n/N (%)*	355/1947 (18.2)	377/1955 (19.3)	0.96 (0.83 to 1.11)
NDD			
*Any MACE, n/N (%)*	382/1739 (22.0)	344/1732 (19.9)	1.17 (1.01 to 1.36)

In the INNO_2_VATE and PRO_2_TECT programs, the primary safety end point was the first occurrence of an adjudicated major adverse cardiovascular event (all-cause mortality, nonfatal myocardial infarction or stroke). CI, confidence interval; DD-CKD, dialysis-dependent CKD; HR, hazard ratio; MACE, major adverse cardiovascular event; NDD-CKD, non–dialysis-dependent CKD; US, United States.

In the pooled analyses of INNO_2_VATE and PRO_2_TECT for the non-US subgroup, MACE occurred in 268/1645 patients (16%) randomized to vadadustat and 256/1644 patients (16%) randomized to darbepoetin alfa (HR, 1.12; 95% CI, 0.94 to 1.33; Figures 1 and 2B and Table 2). In the non-US subgroup of the INNO_2_VATE trials, first MACE occurred in 90/767 (12%) patients randomized to vadadustat and 108 of 774 (14%) patients randomized to darbepoetin alfa (HR, 0.88; 95% CI, 0.67 to 1.17; Figures 1 and 2D and Table 2); In the non-US subgroup of the pooled PRO_2_TECT trials, first MACE occurred in 178/878 (20%) non-US patients randomized to vadadustat group and 148/870 (17%) patients randomized to darbepoetin alfa (HR, 1.29; 95% CI, 1.03 to 1.60; (Figures 1 and 2F and Table 2).

### Change in Hemoglobin

Vadadustat was noninferior to darbepoetin alfa for hemoglobin efficacy in US and non-US patients in the INNO_2_VATE DD-CKD and PRO_2_TECT NDD-CKD programs throughout the trial periods (Supplemental Figure 4 and Supplemental Table 2).

In the INNO_2_VATE trials, the least squares (LS) mean difference in hemoglobin change from baseline to weeks 24–36 (vadadustat versus darbepoetin alfa) was −0.3 g/dl (95% CI, −0.6 to −0.1) and −0.2 g/dl (95% CI, −0.3 to −0.1) in US patients enrolled in the incident and prevalent DD-CKD trials, respectively. The LS mean difference in hemoglobin change from baseline to weeks 24–36 (vadadustat versus darbepoetin alfa) was −0.3 g/dl (95% CI, −0.6 to 0.1) and −0.1 g/dl (95% CI, −0.2 to 0.02) in non-US patients enrolled in the incident and prevalent DD-CKD trials, respectively. Similar results were observed during the secondary evaluation period (weeks 40–52) of both the US and non-US subgroups (Supplemental Table 2).

In the PRO_2_TECT trials, the LS mean difference in hemoglobin change from baseline to weeks 24–36 (vadadustat versus darbepoetin alfa) was 0.2 g/dl (95% CI, 0.04 to 0.3) and 0.02 g/dl (95% CI, −0.1 to 0.2) in US patients enrolled in the ESA-untreated and ESA-treated NDD-CKD trials, respectively. The LS mean difference in hemoglobin change from baseline to weeks 24–36 (vadadustat versus darbepoetin alfa) was −0.1 g/dl (95% CI, −0.3 to 0.1) and −0.03 g/dl (95% CI, −0.1 to 0.1) in non-US patients enrolled in the ESA-untreated and ESA-treated NDD-CKD trials, respectively. Similar results were seen in the secondary evaluation period (weeks 40–52) of both the US and non-US subgroups (Supplemental Table 2).

The mean change in hemoglobin concentrations over time was similar in the two treatment groups in US and non-US patients across all four trials (Supplemental Figure 4). Of note, in US and non-US patients in the vadadustat-treated group in the prevalent DD-CKD trial, there was a transient decrease in mean hemoglobin levels from weeks 2–10, which then gradually rose over time (Supplemental Figure 4, B and F).

The proportions of patients achieving target hemoglobin concentrations of 10–11 g/dl in the United States and 10–12 g/dl outside of the United States during weeks 24–36 and weeks 40–52 were generally similar between treatment groups for the four trials across both evaluation periods (Supplemental Table 3).

### AEs

For the US and non-US patients across all four global phase 3 trials, the incidence of treatment-emergent AEs and SAEs was similar in patients randomized to vadadustat and darbepoetin alfa (Table 3). In the United States, treatment-emergent AEs of special interest (AESI) occurred in 46% of vadadustat-treated and 51% of darbepoetin alfa–treated patients (Table 4), corresponding to a relative risk of 0.90 (95% CI, 0.84 to 0.96). Outside the United States, treatment-emergent AESIs occurred in 35% of vadadustat-treated and 38% of darbepoetin alfa–treated patients, corresponding to a relative risk of 0.93 (95% CI, 0.85 to 1.01; Table 4). In both the US and non-US regions, hypertension, congestive heart failure, and hyperkalemia were the most common AESIs in both treatment groups (Table 4).

**Table 3:** Adverse events in US and non-US patients from the pooled safety populations of the INNO_2_VATE (dialysis-dependent CKD) and PRO_2_TECT (non–dialysis-dependent CKD) trials

Event, *n* (%)	US		Non-US	
	Vadadustat (*n*=2041), PY=3883	Darbepoetin Alfa (*n*=2043), PY=3912	Vadadustat (*n*=1645), PY=2453	Darbepoetin Alfa (*n*=1644), PY=2508
Any TEAEs	1840 (90)	1858 (91)	1437 (87)	1434 (87)
Any drug-related TEAEs	188 (9)	91 (5)	183 (11)	83 (5)
Any severe TEAEs	1055 (52)	1065 (52)	527 (32)	531 (32)
Any treatment-emergent SAEs	1341 (66)	1374 (67)	798 (49)	812 (49)
Any drug-related treatment-emergent SAEs	33 (2)	35 (2)	33 (2)	20 (1)
Any TEAEs leading to study drug discontinuation	158 (8)	96 (5)	101 (6)	30 (2)
Any drug-related TEAEs leading to study drug discontinuation	37 (2)	8 (0.4)	36 (2)	3 (0.2)
Any TEAEs resulting in death	353 (17)	369 (18)	240 (15)	227 (14)
Any deaths	367 (18)	388 (19)	243 (15)	229 (14)
**Common AE >10%^a^**				
Hypertension	306 (15)	361 (18)	189 (12)	225 (14)
Kidney failure	316 (16)	331 (16)	242 (15)	232 (14)
Hyperkalemia	256 (13)	305 (15)	101 (6)	117 (7)
Diarrhea	312 (15)	244 (12)	177 (11)	115 (7)
Fall	265 (13)	276 (14)	49 (3)	44 (3)
Pneumonia	260 (13)	227 (11)	120 (7)	119 (7)
Fluid overload	240 (12)	225 (11)	36 (2)	50 (3)
Urinary tract infection	222 (11)	224 (11)	117 (7)	138 (8)
Nausea	227 (11)	188 (9)	97 (6)	88 (5)

AE, adverse event; PY, patient-years; SAE, serious adverse event; TEAE, treatment-emergent adverse event; US, United States.

a Common adverse events (by preferred term) were those that occurred in >10% of patients in either treatment group.

**Table 4:** Adverse events of special interest in US and non-US patients from the pooled safety populations of the INNO_2_VATE (dialysis-dependent CKD) and PRO_2_TECT (non–dialysis-dependent CKD) trials

Event, *n* (%)	US			Non-US		
	Vadadustat (*n*=2041), PY=3883	Darbepoetin Alfa (*n*=2043), PY=3912	Vadadustat/Darbepoetin Alfa, Relative Risk (95% CI)	Vadadustat (*n*=1645), PY=2453	Darbepoetin Alfa (*n*=1644), PY=2508	Vadadustat/Darbepoetin Alfa, Relative Risk (95% CI)
Any TEAEs of special interest	942 (46)	1050 (51)	0.90 (0.84 to 0.96)	572 (35)	617 (38)	0.93 (0.85 to 1.01)
**AESIs (by preferred term)**						
Hypertension	408 (20)	477 (23)	0.86 (0.76 to 0.96)	255 (16)	299 (18)	0.85 (0.73 to 0.99)
Congestive heart failure	276 (14)	316 (16)	0.87 (0.75 to 1.02)	105 (6)	109 (7)	0.96 (0.74 to 1.25)
Hyperkalemia	259 (13)	316 (16)	0.82 (0.70 to 0.96)	105 (6)	123 (8)	0.85 (0.66 to 1.10)
Hypersensitivity	190 (9)	192 (9)	0.99 (0.82 to 1.2)	92 (6)	99 (6)	0.93 (0.71 to 1.22)
Hepatotoxicity	157 (8)	164 (8)	0.96 (0.78 to 1.18)	95 (6)	75 (5)	1.27 (0.94 to 1.70)
Malignant or unspecified tumors	77 (4)	97 (5)	0.8 (0.6 to 1.06)	44 (3)	52 (3)	0.85 (0.57 to 1.26)
Pulmonary hypertension	76 (4)	82 (4)	0.93 (0.68 to 1.26)	11 (1)	13 (1)	0.85 (0.38 to 1.88)
Cardiac valve disorders	60 (3)	63 (3)	0.95 (0.67 to 1.35)	24 (2)	21 (1)	1.14 (0.64 to 2.04)
Retinal effects	43 (2)	37 (2)	1.16 (0.75 to 1.8)	31 (2)	42 (3)	0.74 (0.47 to 1.17)
Adrenal disorder	3 (0.1)	3 (0.1)	1.0 (0.2 to 4.95)	1 (0.1)	0	—

AESI, adverse events of special interest; CI, confidence interval; PY, patient-years; TEAE, treatment-emergent adverse event; US, United States.

## Discussion

The current analysis summarizes safety and efficacy of vadadustat compared with darbepoetin alfa in US and non-US patients enrolled in the INNO_2_VATE and PRO_2_TECT programs. The US subgroup made up most patients in the global trials and is of interest because of distinct prescribing patterns and near-universal access to life-sustaining dialysis treatment with the development of kidney failure—a feature not uniformly available in all countries. Notably, in the global INNO_2_VATE and PRO_2_TECT populations (both US and non-US), MACE risk remained similar between treatment groups in patients with DD-CKD, while vadadustat did not meet the criteria for noninferiority with respect to MACE in patients in the NDD-CKD trials.^12,13^ In US patients, the relative risk of MACE (comparing vadadustat with darbepoetin alfa) was similar in the INNO_2_VATE DD-CKD program and the PRO_2_TECT NDD-CKD program.

The non-US PRO_2_TECT subgroup revealed a higher risk of MACE associated with the use of vadadustat. We have previously investigated the trials in patients previously treated and not treated with ESAs separately and uncovered several factors within these trials that may have influenced the results.^18,19^ *Post hoc* analyses of the PRO_2_TECT trial in ESA-untreated patients in some countries outside of the United States and Europe noted exceedingly high MACE rates in the vadadustat treatment group; we noted that randomization was imbalanced with respect to baseline eGFR in two countries (Brazil and South Africa) with high rates of enrollment and where only a small fraction of patients initiated dialysis. In these countries, there was a larger proportion of patients with an eGFR of <10 ml/min per 1.73 m^2^ randomized to vadadustat (40%) compared with darbepoetin alfa (23%). Many patients in these countries died of noncardiovascular death, which was primarily attributed to kidney failure.^18^ Moreover, in the ESA-treated trial, while clinical characteristics were reasonably well balanced at baseline, we noted that MACE rates were roughly half of those expected among patients in Europe randomized to darbepoetin alfa. Conversely, data from US patients with NDD-CKD indicated similar risks of MACE whether randomized to vadadustat or darbepoetin alfa.^19^

In both the US and non-US patient subgroups, vadadustat was noninferior to darbepoetin alfa in correcting and maintaining hemoglobin concentrations within the target range (US: 10–11 g/dl; non-US: 10–12 g/dl). Of note, the initial transient decline in hemoglobin levels observed in the US and non-US subgroups of the prevalent INNO_2_VATE trial mirrors that observed in the global study, presumably due to trial design and the need for higher starting doses in some patients.^12^

Clinical trials requiring large sample sizes often necessitate recruitment from multiple countries, and demonstrating consistent benefits across multiple populations can instill confidence in patients and care providers when prescribing new medications. However, conducting global trials poses significant challenges, particularly where clinical landscapes differ across regions, as is the case in CKD-related anemia.^20^ For instance, it is more common for older patients to pursue dialysis in the United States than in some other countries. Demographic factors such as the proportion of patients of African ancestry and dietary considerations, including access to and consumption habits regarding red meat, exhibit significant variation across different regions. The use of peritoneal dialysis rather than hemodialysis varies significantly, with the latter often associated with regular blood loss. There is also wide variation in the types of vascular access used for hemodialysis around the world; for instance, hemodialysis through a central venous catheter (with associated inflammation and relative resistance to ESAs) is much more common in the United States than elsewhere. Moreover, clinical practice guidelines and standards regarding hemoglobin targets exhibit regional discrepancies. Furthermore, there is variation in the relative use of oral and intravenous iron, ESAs, and blood transfusion. Therefore, performing focused analyses by region can be beneficial, even if statistical power may be compromised.

Yusuf and Wittes^20^ provided an extremely thoughtful report on interpreting geographic differences in results from randomized controlled trials, citing several examples of disparate results within and outside of the United States, including the cardiovascular outcome trials Metoprolol CR/XL Randomized Intervention Trial in Congestive Heart Failure,^21^ Platelet Inhibition and Patient Outcomes,^22^ and Treatment of Preserved Cardiac Function Heart Failure with an Aldosterone Antagonist Trial.^23,24^ They concluded, “When a randomized, clinical trial shows marked variations in results among countries, one should seek supporting evidence to understand whether the observed results are likely to be real, an artifact of the design analysis or implementation of the trial, or simply due to chance.”^20^ In the case of the PRO_2_TECT program, the current analysis follows this recommendation. Moreover, we consider this subanalysis relevant due to the considerable proportion of US participants in the PRO_2_TECT trials. Of the 3471 patients randomized, 1723 participants (50%) were from the United States.

The present analysis benefits from the diversity of the US patient population for age, sex, race and ethnicity, and causes of underlying kidney disease. Although the sample sizes within the US cohort were substantial, they were not adequate in either INNO_2_VATE or PRO_2_TECT to reliably determine noninferiority with respect to cardiovascular safety with the 1.25 margin selected for the overall trial.^12,13^ The analysis was constrained by the inclusion of many patients already receiving darbepoetin at the start of the trials. Consequently, these individuals were predisposed to better tolerate and respond to darbepoetin than patients treated with an alternative active control agent. Although a placebo control could have mitigated that bias, it would have been unacceptable to most patients and clinicians, particularly for patients with DD-CKD.

In sum, among patients with DD-CKD, safety (vis-à-vis MACE) and efficacy (vis-à-vis change in hemoglobin) of vadadustat and darbepoetin alfa were similar when stratified by region (US versus non-US). In US patients with NDD-CKD, safety of vadadustat and darbepoetin alfa were similar; outside the United States, there was a higher relative risk for MACE with vadadustat, a finding that could be incidental or may reflect differences in patient characteristics, hemoglobin targets or other practice patterns, or access to health care services, including dialysis.

## Supplementary Material

SUPPLEMENTARY MATERIAL

## Data Availability

Partial restrictions to the data and/or materials apply. Proposal requests for access to original data should be sent to medicalinfo@akebia.com. Deidentified patient-level data will be available 12 months after US and European Union approval to qualified researchers with an appropriate research proposal. The research proposal is subject to review by an independent review board with final approval by Akebia Therapeutics, Inc.

## Supplemental Material

Supplemental Table 1. Locations of the INNO_2_VATE and PRO_2_TECT trials.

Supplemental Table 2. Change in hemoglobin from baseline in US and non-US patients from the INNO_2_VATE (DD-CKD) and PRO_2_TECT (NDD-CKD) trials.

Supplemental Table 3. Proportion of patients with average hemoglobin values within the US-specific or non–US-specific target range during weeks 24–36 and weeks 40–52 (stratified Mantel–Haenszel method with observed data).

Supplemental Figure 1. INNO_2_VATE (DD-CKD) and PRO_2_TECT (NDD-CKD) trial design.

Supplemental Figure 2. Patient disposition.

Supplemental Figure 3. MACE and MACE-related outcomes in US patients from the INNO_2_VATE (DD-CKD) and PRO_2_TECT (NDD-CKD) trials, adjusted for age as a continuous variable.

Supplemental Figure 4. Mean change in hemoglobin from baseline over time for US and non-US patients from the INNO_2_VATE (DD-CKD) and PRO_2_TECT (NDD-CKD) trials.
