# Supplementary material for: Safety and Efficacy of Vadadustat for the Treatment of CKD-Related Anemia within and outside the United States
Source: J Am Soc Nephrol. 2025 May 13;36(10):1984–97. doi: 10.1681/ASN.0000000708 (PMC12499611; doi:10.1681/ASN.0000000708)
Supplement: SUPPLEMENTARY MATERIAL [file jasn-36-1984-s001.pdf]

ASN
American Society of Nephrology
JASN
Journal of the American Society of Nephrology
KIDNEY360
Accessing Our World From Every Angle
CJASN
Clinical Journal of the American Society of Nephrology

ASN Journal Disclosure Form

As per ASN journal policy, I have disclosed any financial relationships or commitments I have held in the past 36 months as included below. I have listed my Current Employer below to indicate there is a relationship requiring disclosure. If no relationship exists, my Current Employer is not listed.

R. Agarwal reports the following:
Employer: Veteran's Administration (part time); Consultancy: Akebia, Bayer, Boehringer Ingelheim, Eli Lilly, Chinook, Alnylam, Vertex, Intercept Pharmaceuticals, Novartis; Honoraria: Akebia, Bayer, Boehringer Ingelheim, Eli Lilly, Chinook, Vertex, Intercept Pharmaceuticals, Eloxx, Novartis, Alnylam; Patents or Royalties: UpToDate; and Advisory or Leadership Role: KDIGO, Hypertension, NDT, JASH, Seminars in Dialysis, Akebia, Bayer, Boehringer Ingelheim, Eli Lilly, Chinook, Diamedica, Vertex, Eloxx, Novartis, Alnylam.

I understand that the information above will be published within the journal article, if accepted, and that failure to comply and/or to accurately and completely report the potential financial conflicts of interest could lead to the following: 1) Prior to publication, article rejection, or 2) Post-publication, sanctions ranging from, but not limited to, issuing a correction, reporting the inaccurate information to the authors' institution, banning authors from submitting work to ASN journals for varying lengths of time, and/or retraction of the published work.

Name: Rajiv Agarwal
Manuscript ID: JASN-2024-001247R1
Manuscript Title: Safety and efficacy of Vadadustat....
Date of Completion: March 5, 2025
Disclosure Updated Date: October 3, 2024

ASN
American Society of Nephrology
JASN
Journal of the American Society of Nephrology
KIDNEY360
Accessing Our World From Every Angle
CJASN
Clinical Journal of the American Society of Nephrology

ASN Journal Disclosure Form

As per ASN journal policy, I have disclosed any financial relationships or commitments I have held in the past 36 months as included below. I have listed my Current Employer below to indicate there is a relationship requiring disclosure. If no relationship exists, my Current Employer is not listed.

S. Burke reports the following:
Employer: Akebia Therapeutics; Consultancy: Endomimetics; Ownership Interest: Akebia Therapeutics; Patents or Royalties: Pharmacosmos; and Other Interests or Relationships: American Kidney Fund fundraising event annually; Kidney Health Initiative board member (drugs committee).

I understand that the information above will be published within the journal article, if accepted, and that failure to comply and/or to accurately and completely report the potential financial conflicts of interest could lead to the following: 1) Prior to publication, article rejection, or 2) Post-publication, sanctions ranging from, but not limited to, issuing a correction, reporting the inaccurate information to the authors' institution, banning authors from submitting work to ASN journals for varying lengths of time, and/or retraction of the published work.

Name: Steven K. Burke
Manuscript ID: JASN-2024-001247R1
Manuscript Title: Safety and Efficacy of Vadadustat for the Treatment of CKD-Related Anemia Within and Outside the United States
Date of Completion: February 21, 2025
Disclosure Updated Date: February 21, 2025

ASN American Society of Nephrology | JASN Journal of the American Society of Nephrology | KIDNEY360 Accessing Our World From Every Angle | CJASN Clinical Journal of the American Society of Nephrology

ASN Journal Disclosure Form

As per ASN journal policy, I have disclosed any financial relationships or commitments I have held in the past 36 months as included below. I have listed my Current Employer below to indicate there is a relationship requiring disclosure. If no relationship exists, my Current Employer is not listed.

G. Chertow reports the following:
Employer: Stanford University School of Medicine; Consultancy: Akebia, Alebund, Ardelyx, AstraZeneca, CalciMedica, Miromatrix, Panoramic, Sanifit, Toku, Unicycive, Vertex; Ownership Interest: Ardelyx, CloudCath, Durect, Eliaz Therapeutics, Outset, Renibus, Unicycive; Research Funding: NIDDK, NIAID, CSL Behring; Advisory or Leadership Role: Board of Directors, Satellite Healthcare, Co-Editor, Brenner & Rector's The Kidney (Elsevier); and Other Interests or Relationships: DSMB service: NIDDK, George Institute, Aethlon, Bayer, Mineralys, ReCor.

I understand that the information above will be published within the journal article, if accepted, and that failure to comply and/or to accurately and completely report the potential financial conflicts of interest could lead to the following: 1) Prior to publication, article rejection, or 2) Post-publication, sanctions ranging from, but not limited to, issuing a correction, reporting the inaccurate information to the authors' institution, banning authors from submitting work to ASN journals for varying lengths of time, and/or retraction of the published work.

Name: Glenn M. Chertow
Manuscript ID: JASN-2024-001247R1
Manuscript Title: Safety and Efficacy of Vadadustat for the Treatment of CKD-Related Anemia Within and Outside the United States
Date of Completion: February 27, 2025
Disclosure Updated Date: January 22, 2025

ASN American Society of Nephrology | JASN Journal of the American Society of Nephrology | KIDNEY360 Accessing Our World From Every Angle | CJASN Clinical Journal of the American Society of Nephrology

ASN Journal Disclosure Form

As per ASN journal policy, I have disclosed any financial relationships or commitments I have held in the past 36 months as included below. I have listed my Current Employer below to indicate there is a relationship requiring disclosure. If no relationship exists, my Current Employer is not listed.

K. Eckardt reports the following:
Employer: Charité - Universitätsmedizin Berlin; Consultancy: Akebia, Astra Zeneca, Boehringer CLS Behring, Ingelheim, GSK, Medice, Novartis; Research Funding: Evotec, Travere; Honoraria: Akebia, Astra Zeneca, Bayer, Boehringer Ingelheim, CLS Behring, GSK, Medice, Novartis; and Advisory or Leadership Role: Editorial Board: KI.

I understand that the information above will be published within the journal article, if accepted, and that failure to comply and/or to accurately and completely report the potential financial conflicts of interest could lead to the following: 1) Prior to publication, article rejection, or 2) Post-publication, sanctions ranging from, but not limited to, issuing a correction, reporting the inaccurate information to the authors' institution, banning authors from submitting work to ASN journals for varying lengths of time, and/or retraction of the published work.

Name: Kai-Uwe Eckardt
Manuscript ID: JASN-2024-001247R1
Manuscript Title: Safety and Efficacy of Vadadustat for the Treatment of CKD-Related Anemia Within and Outside the United States
Date of Completion: February 15, 2025
Disclosure Updated Date: November 19, 2024

ASN
American Society of Nephrology
JASN
Journal of the American Society of Nephrology
KIDNEY360
Accessing Our World From Every Angle
CJASN
Clinical Journal of the American Society of Nephrology

ASN Journal Disclosure Form

As per ASN journal policy, I have disclosed any financial relationships or commitments I have held in the past 36 months as included below. I have listed my Current Employer below to indicate there is a relationship requiring disclosure. If no relationship exists, my Current Employer is not listed.

W. Luo reports the following:
Employer: Akebia Therapeutics, Inc.;; and Ownership Interest: Akebia Therapeutics, Inc.;.

I understand that the information above will be published within the journal article, if accepted, and that failure to comply and/or to accurately and completely report the potential financial conflicts of interest could lead to the following: 1) Prior to publication, article rejection, or 2) Post-publication, sanctions ranging from, but not limited to, issuing a correction, reporting the inaccurate information to the authors' institution, banning authors from submitting work to ASN journals for varying lengths of time, and/or retraction of the published work.

Name: Wenli Luo
Manuscript ID: JASN-2024-001247R1
Manuscript Title: Safety and Efficacy of Vadadustat for the Treatment of CKD-Related Anemia Within and Outside the United States
Date of Completion: February 18, 2025
Disclosure Updated Date: February 18, 2025

ASN American Society of Nephrology
JASN Journal of the American Society of Nephrology
KIDNEY360 Accessing Our World From Every Angle
CJASN Clinical Journal of the American Society of Nephrology

ASN Journal Disclosure Form

As per ASN journal policy, I have disclosed any financial relationships or commitments I have held in the past 36 months as included below. I have listed my Current Employer below to indicate there is a relationship requiring disclosure. If no relationship exists, my Current Employer is not listed.

T. Minga reports the following:
Employer: Akebia Therapeutics; Maze Therapeutics; and Ownership Interest: Akebia Therapeutics; Maze Therapeutics.

I understand that the information above will be published within the journal article, if accepted, and that failure to comply and/or to accurately and completely report the potential financial conflicts of interest could lead to the following: 1) Prior to publication, article rejection, or 2) Post-publication, sanctions ranging from, but not limited to, issuing a correction, reporting the inaccurate information to the authors' institution, banning authors from submitting work to ASN journals for varying lengths of time, and/or retraction of the published work.

Name: Todd Eric Minga
Manuscript ID: JASN-2024-001247R1
Manuscript Title: Safety and Efficacy of Vadadustat for the Treatment of CKD-Related Anemia Within and Outside the United States
Date of Completion: February 18, 2025
Disclosure Updated Date: February 18, 2025

ASN American Society of Nephrology | JASN Journal of the American Society of Nephrology | KIDNEY360 Accessing Our World From Every Angle | CJASN Clinical Journal of the American Society of Nephrology

ASN Journal Disclosure Form

As per ASN journal policy, I have disclosed any financial relationships or commitments I have held in the past 36 months as included below. I have listed my Current Employer below to indicate there is a relationship requiring disclosure. If no relationship exists, my Current Employer is not listed.

M. Sarnak reports the following:
Employer: My spouse works for Eli Lilly; Consultancy: Steering Committee of Trials Funded by Akebia; Ownership Interest: spouse is employee of Eli Lilly; and Research Funding: NIH.

I understand that the information above will be published within the journal article, if accepted, and that failure to comply and/or to accurately and completely report the potential financial conflicts of interest could lead to the following: 1) Prior to publication, article rejection, or 2) Post-publication, sanctions ranging from, but not limited to, issuing a correction, reporting the inaccurate information to the authors' institution, banning authors from submitting work to ASN journals for varying lengths of time, and/or retraction of the published work.

Name: Mark J. Sarnak
Manuscript ID: JASN-2024-001247R1
Manuscript Title: Safety and Efficacy of Vadadustat for the Treatment of CKD-Related Anemia Within and Outside the United States
Date of Completion: February 26, 2025
Disclosure Updated Date: February 26, 2025

ASN
American Society of Nephrology
JASN
Journal of the American Society of Nephrology
KIDNEY360
Accessing Our World From Every Angle
CJASN
Clinical Journal of the American Society of Nephrology

ASN Journal Disclosure Form

As per ASN journal policy, I have disclosed any financial relationships or commitments I have held in the past 36 months as included below. I have listed my Current Employer below to indicate there is a relationship requiring disclosure. If no relationship exists, my Current Employer is not listed.

W. Winkelmayer reports the following:
Employer: Baylor College of Medicine; Consultancy: Akebia, Anthos, AstraZeneca, Bayer, Boehringer Ingelheim, Cadrenal, GlaxoSmithKline, Idorsia, Merck, Natera, Novartis, Pharmacosmos, Vera.; Honoraria: Akebia, Anthos, AstraZeneca, Bayer, Boehringer Ingelheim, Cadrenal, GlaxoSmithKline, Idorsia, Merck, Natera, Novartis, Pharmacosmos, Vera.; and Advisory or Leadership Role: Journal of the American Medical Association (Associate Editor).

I understand that the information above will be published within the journal article, if accepted, and that failure to comply and/or to accurately and completely report the potential financial conflicts of interest could lead to the following: 1) Prior to publication, article rejection, or 2) Post-publication, sanctions ranging from, but not limited to, issuing a correction, reporting the inaccurate information to the authors' institution, banning authors from submitting work to ASN journals for varying lengths of time, and/or retraction of the published work.

Name: Wolfgang C. Winkelmayer
Manuscript ID: JASN-2024-001247R1
Manuscript Title: Safety and Efficacy of Vadadustat for the Treatment of CKD-Related Anemia Within and Outside the United States
Date of Completion: February 17, 2025
Disclosure Updated Date: November 22, 2024
