# Supplementary material for: Safety and Efficacy of Vadadustat for the Treatment of CKD-Related Anemia within and outside the United States
Source: J Am Soc Nephrol. 2025 May 13;36(10):1984–97. doi: 10.1681/ASN.0000000708 (PMC12499611; doi:10.1681/ASN.0000000708)
Supplement: SUPPLEMENTARY MATERIAL [file jasn-36-1984-s002.pdf]

Supplemental Material for:

Safety and Efficacy of Vadadustat for the Treatment of CKD-Related Anemia Within and Outside the United States

GM Chertow, et al.

**Supplemental Material Table of Contents**	**Supplemental Page**
Supplemental Table 1. Locations of the INNO$_2$VATE and PRO$_2$TECT Trials	1
Supplemental Table 2. Change in Hemoglobin From Baseline in US and Non-US Patients From the INNO$_2$VATE (DD-CKD) and PRO$_2$TECT (NDD-CKD) Trials	2
Supplemental Table 3. Proportion of Patients With Average Hemoglobin Values Within the US- or Non-US-Specific Target Range During Weeks 24-36 and Weeks 40-52 (Stratified Mantel-Haenszel Method With Observed Data)	4
Supplemental Figure 1. INNO$_2$VATE (DD-CKD) and PRO$_2$TECT (NDD-CKD) Trial Design	6
Supplemental Figure 2. Patient Disposition	7
Supplemental Figure 3. MACE and MACE-Related Outcomes in US Patients From the INNO$_2$VATE (DD-CKD) and PRO$_2$TECT (NDD-CKD) Trials, Adjusted for Age as a Continuous Variable	8
Supplemental Figure 4. Mean Change in Hemoglobin From Baseline Over Time for US and Non-US Patients From the INNO$_2$VATE (DD-CKD) and PRO$_2$TECT (NDD-CKD) Trials	9

Supplemental Table 1. Locations of the INNO$_2$VATE and PRO$_2$TECT Trials

INNO$_2$VATE DD-CKD Trials	PRO$_2$TECT NDD-CKD Trials
Argentina	Argentina
Australia	Australia
Brazil	Austria
Bulgaria	Brazil
Canada	Bulgaria
France	Canada
Germany	Chile
Israel	Colombia
Italy	Czech Republic
Mexico	France
Poland	Germany
Portugal	Hungary
Republic of Korea	Israel
Russia	Italy
Serbia	Republic of Korea
United Kingdom	Malaysia
Ukraine	Mexico
United States of America	Romania
	Russia
	Serbia
	Slovakia
	South Africa
	Spain
	Turkey
	Ukraine
	United Kingdom
	United States of America

CKD, chronic kidney disease; DD-CKD, dialysis-dependent CKD; NDD-CKD, non-dialysis dependent CKD.

Supplemental Table 2. Change in Hemoglobin From Baseline in US and Non-US Patients From the INNO$_2$VATE (DD-CKD) and PRO$_2$TECT (NDD-CKD) Trials

Hemoglobin	US		Non-US	
	Vadadustat	Darbepoetin Alfa	Vadadustat	Darbepoetin Alfa
**INNO$_2$VATE DD-CKD Trials**				
Incident dialysis				
Hemoglobin, g/dL, mean (SD) at weeks 24–36	10.1 (1.0)	10.5 (0.9)	10.6 (1.2)	10.8 (0.9)
Treatment comparison, vadadustat–darbepoetin alfa, LS mean difference in hemoglobin change from baseline (95% CI)	–0.3 (–0.6, –0.1)		–0.3 (–0.6, 0.1)	
Hemoglobin, g/dL, mean (SD) at weeks 40–52	10.5 (1.2)	10.4 (1.1)	10.6 (1.2)	10.7 (1.1)
Treatment comparison, vadadustat–darbepoetin alfa, LS mean difference in hemoglobin change from baseline (95% CI)	0.03 (–0.3, 0.4)		–0.2 (–0.6, 0.2)	
Prevalent dialysis				
Hemoglobin, g/dL, mean (SD) at weeks 24–36	10.2 (0.9)	10.4 (0.9)	10.7 (1.0)	10.7 (1.0)
Treatment comparison, vadadustat–darbepoetin alfa, LS mean difference in hemoglobin change from baseline (95% CI)	–0.2 (–0.3, –0.1)		–0.1 (–0.2, 0.02)	
Hemoglobin, g/dL, mean (SD) at weeks 40–52	10.2 (1.0)	10.4 (0.9)	10.7 (1.1)	10.9 (1.0)
Treatment comparison, vadadustat–darbepoetin alfa, LS mean difference in hemoglobin change from baseline (95% CI)	–0.2 (–0.3, –0.1)		–0.2 (–0.3, –0.1)	
**PRO$_2$TECT NDD-CKD Trials**				
ESA-untreated				
Hemoglobin, g/dL, mean (SD) at weeks 24–36	10.3 (0.9)	10.2 (0.9)	10.5 (1.2)	10.6 (1.2)
Treatment comparison, vadadustat–darbepoetin alfa, LS mean difference in hemoglobin change from baseline (95% CI)	0.2 (0.04, 0.3)		–0.1 (–0.3, 0.1)	
Hemoglobin, g/dL, mean (SD) at weeks 40–52	10.4 (0.9)	10.3 (0.9)	10.6 (1.2)	10.7 (1.1)

Treatment comparison, vadadustat–darbepoetin alfa, LS mean difference in hemoglobin change from baseline (95% CI)	0.1 (–0.02, 0.2)		–0.1 (–0.2, 0.1)	
ESA-treated				
Hemoglobin, g/dL, mean (SD) at weeks 24–36	10.2 (0.9)	10.2 (0.8)	11.1 (0.9)	11.1 (0.9)
Treatment comparison, vadadustat–darbepoetin alfa, LS mean difference in hemoglobin change from baseline (95% CI)	0.02 (–0.1, 0.2)		–0.03 (–0.1, 0.1)	
Hemoglobin, g/dL, mean (SD) at weeks 40–52	10.3 (0.9)	10.2 (0.9)	11.1 (1.0)	11.2 (1.0)
Treatment comparison, vadadustat–darbepoetin alfa, LS mean difference in hemoglobin change from baseline (95% CI)	0.1 (–0.1, 0.2)		–0.04 (–0.2, 0.1)	

The prespecified noninferiority margin for change in hemoglobin levels was a lower bound of −0.75 g/dL.

CKD, chronic kidney disease; DD-CKD, dialysis-dependent CKD; ESA, erythropoiesis-stimulating agent; LS, least squares; NDD-CKD, non-dialysis-dependent CKD; US, United States.

Supplemental Table 3. Proportion of Patients With Average Hemoglobin Values Within the US- or Non-US-Specific Target Range During Weeks 24-36 and Weeks 40-52 (Stratified Mantel-Haenszel Method With Observed Data)

	INNO₂VATE DD-CKD Trials				PRO₂TECT NDD-CKD Trials			
	Incident Dialysis		Prevalent Dialysis		ESA-Untreated		ESA-Treated	
	Vadadustat	Darbepoetin alfa	Vadadustat	Darbepoetin alfa	Vadadustat	Darbepoetin alfa	Vadadustat	Darbepoetin alfa
**Weeks 24-36**								
**US patients**	n=97	n=102	n=1090	n=1086	n=532	n=529	n=330	n=335
Responders based on the observed data, n (%) (95% CI)	33 (34) (25, 44)	46 (45) (35, 55)	450 (41) (38, 44)	514 (47) (44, 50)	251 (47) (43, 52)	233 (44) (40, 48)	147 (45) (39, 50)	151 (45) (40, 51)
Proportion difference (vadadustat – darbepoetin alfa) (95% CI)	–0.11 (–0.25, 0.02)		–0.06 (–0.10, –0.02)		0.03 (–0.03, 0.09)		–0.01 (–0.08, 0.07)	
**Non-US patients**	n=84	n=86	n=687	n=691	n=347	n=343	n=532	n=528
Responders based on the observed data, n (%) (95% CI)	46 (55) (44, 66)	61 (71) (60, 80)	424 (62) (58, 65)	432 (63) (59, 66)	192 (55) (50, 61)	205 (60) (54, 65)	371 (70) (66, 74)	373 (71) (67, 75)
Proportion difference (vadadustat – darbepoetin alfa) (95% CI)	–0.16 (–0.31, –0.02)		–0.01 (–0.06, 0.04)		–0.05 (–0.12, 0.03)		–0.01 (–0.06, 0.05)	
**Weeks 40-52**								
**US patients**	n=97	n=102	n=1090	n=1086	n=532	n=529	n=330	n=335
Responders based on the observed data, n (%) (95% CI)	32 (33) (24, 43)	34 (33) (24, 43)	398 (37) (34, 39)	483 (45) (41, 47)	218 (41) (37, 45)	205 (39) (35, 43)	131 (40) (34, 45)	130 (39) (34, 44)
Proportion difference (vadadustat – darbepoetin alfa) (95% CI)	–0.01 (–0.14, 0.13)		–0.08 (–0.12, –0.04)		0.02 (–0.04, 0.08)		0.01 (–0.07, 0.08)	
**Non-US patients**	n=84	n=86	n=687	n=691	n=347	n=343	n=532	n=528

Responders based on the observed data, n (%) (95% CI)	40 (48) (37, 59)	43 (50) (39, 61)	389 (57) (53, 60)	422 (61) (57, 65)	161 (46) (41, 52)	174 (51) (45, 56)	306 (58) (53, 62)	293 (56) (51, 60)
Proportion difference (vadadustat – darbepoetin alfa) (95% CI)	−0.02 (−0.17, 0.13)		−0.04 (−0.10, 0.01)		−0.04 (−0.12, 0.03)		0.02 (−0.04, 0.08)	

CKD, chronic kidney disease; DD-CKD, dialysis-dependent CKD; NDD-CKD, non-dialysis dependent CKD; ESA, erythropoiesis-stimulating agent; US, United States.

Supplemental Figure 1. INNO$_2$VATE (DD-CKD) and PRO$_2$TECT (NDD-CKD) Trial Design

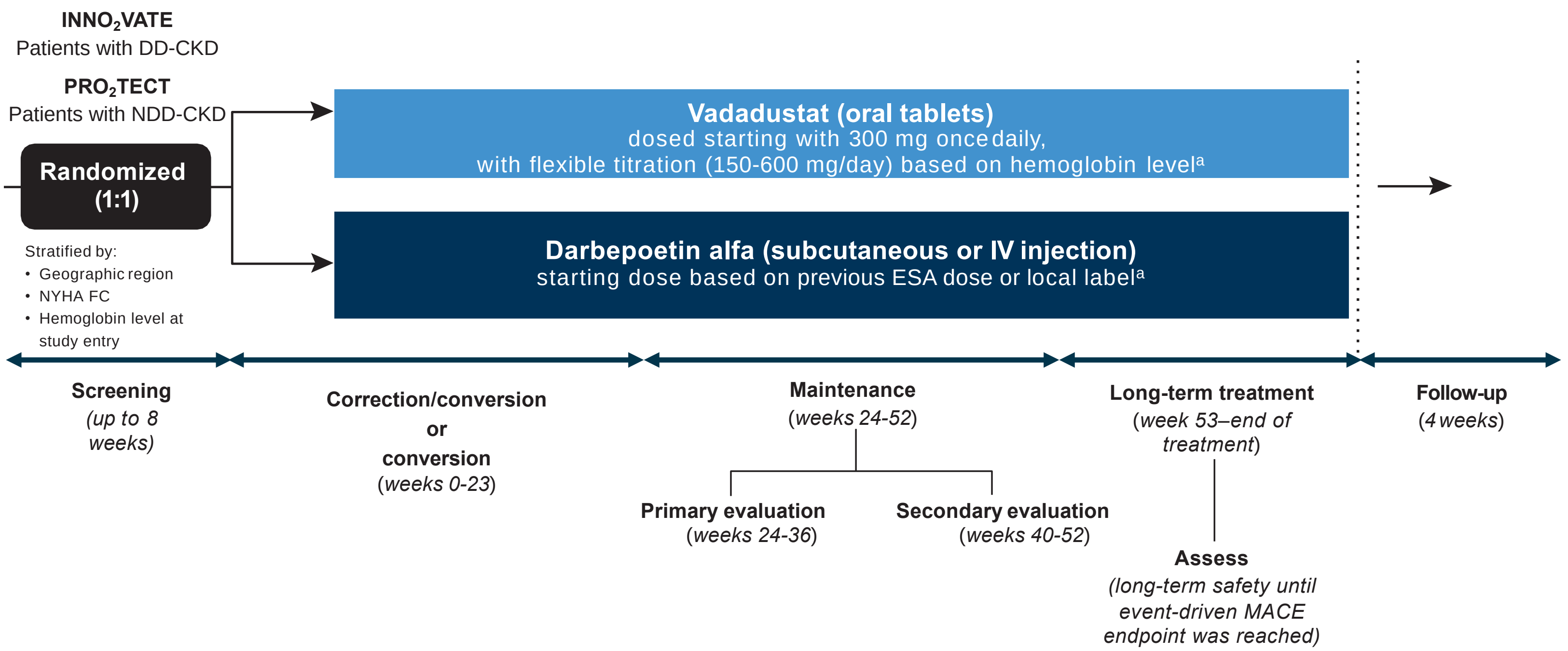

[a]Study drug is titrated to achieve target hemoglobin levels (US: 10-11 g/dL; non-US: 10-12 g/dL).

CKD, chronic kidney disease; DD-CKD, dialysis-dependent CKD; ESA, erythropoiesis-stimulating agent; IV, intravenous; MACE, major adverse cardiovascular events; NDD-CKD, non–dialysis-dependent CKD; NYHA CHF, New York Heart Association Functional Class.

Supplemental Figure 2. Patient Disposition

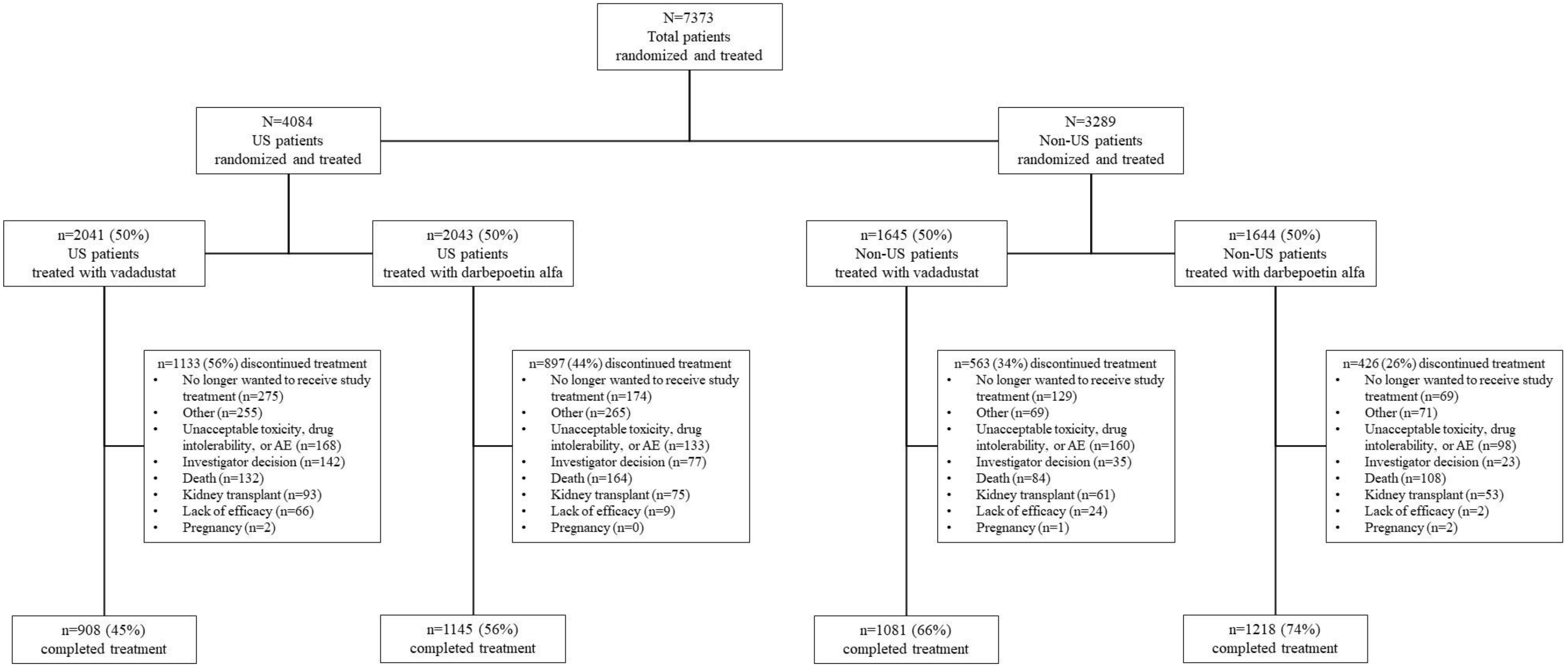

Supplemental Figure 3. MACE and MACE-Related Outcomes in US Patients From the INNO$_2$VATE (DD-CKD) and PRO$_2$TECT (NDD-CKD) Trials, Adjusted for Age as a Continuous Variable

	Vadadustat	Darbepoetin Alfa	Treatment Comparison Vadadustat/Darbepoetin Alfa	HR (95% CI)
**NDD-CKD + DD-CKD**				
**Any MACE, n/N (%)**	469/2041 (23)	465/2043 (23)		1.01 (0.89, 1.15)
**All-cause mortality, n/N (%)**	367/2041 (18)	388/2043 (19)		0.94 (0.81, 1.08)
**Expanded MACE**	578/2041 (28)	584/2043 (29)		1.00 (0.89, 1.12)
**Cardiovascular MACE, n/N (%)**	295/2041 (14)	283/2043 (14)		1.06 (0.90, 1.25)
**Cardiovascular deaths, n/N (%)**	178/2041 (9)	187/2043 (9)		0.96 (0.78, 1.18)
**DD-CKD**				
**Any MACE, n/N (%)**	265/1180 (22)	269/1181 (23)		1.01 (0.85, 1.19)
**All-cause mortality, n/N (%)**	212/1180 (18)	218/1181 (18)		0.98 (0.81, 1.18)
**Expanded MACE**	321/1180 (27)	330/1181 (28)		0.99 (0.85, 1.16)
**Cardiovascular MACE, n/N (%)**	174/1180 (15)	180/1181 (15)		0.99 (0.80, 1.22)
**Cardiovascular deaths, n/N (%)**	112/1180 (9)	117/1181 (10)		0.96 (0.75, 1.25)
**NDD-CKD**				
**Any MACE, n/N (%)**	204/861 (24)	196/862 (23)		1.01 (0.83, 1.23)
**All-cause mortality, n/N (%)**	155/861 (18)	170/862 (20)		0.86 (0.69, 1.07)
**Expanded MACE**	257/861 (30)	254/862 (30)		0.99 (0.83, 1.18)
**Cardiovascular MACE, n/N (%)**	121/861 (14)	103/862 (12)		1.16 (0.89, 1.52)
**Cardiovascular deaths, n/N (%)**	66/861 (8)	70/862 (8)		0.92 (0.65, 1.29)

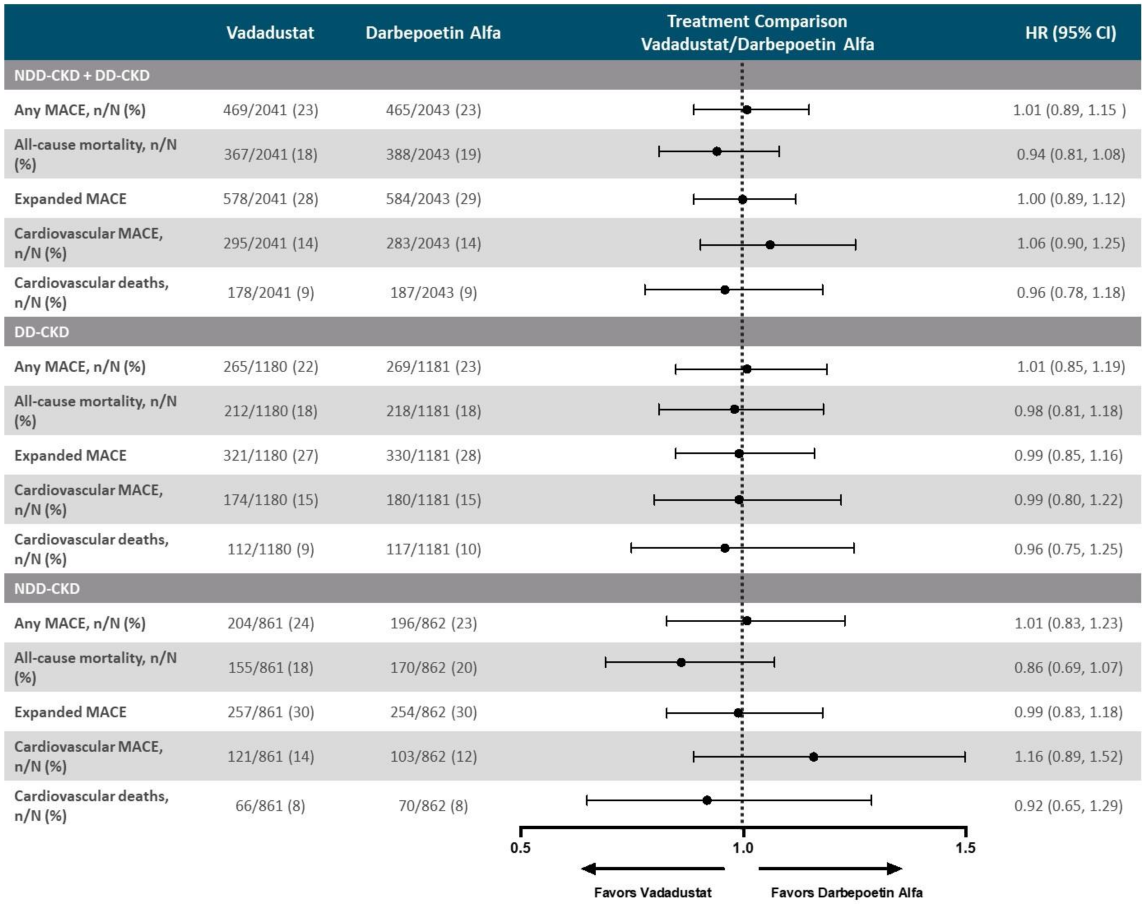

CKD, chronic kidney disease; DD-CKD, dialysis-dependent CKD; HR, hazard ratio; MACE, major adverse cardiovascular event; NDD-CKD, non–dialysis-dependent CKD.

Supplemental Figure 4. Mean Change in Hemoglobin From Baseline Over Time for US and Non-US Patients From the INNO$_2$VATE (DD-CKD) and PRO$_2$TECT (NDD-CKD) Trials

A. Incident Dialysis – US INNO$_2$VATE DD-CKD

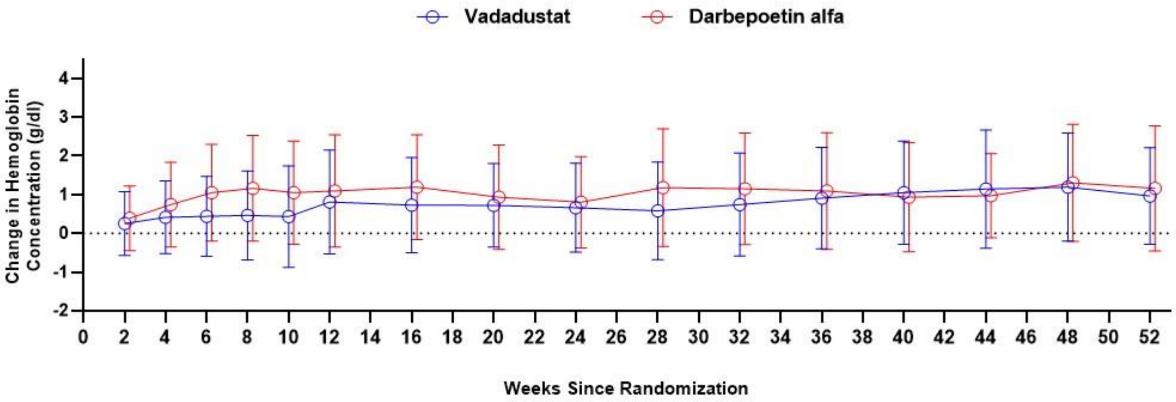

No. at Risk

Vadadustat	89	89	88	87	86	87	85	86	81	81	80	70	67	58	55	47
Darbepoetin alfa	97	90	91	90	86	85	86	87	87	84	78	76	71	53	52	50

B. Prevalent Dialysis - US

INNO$_2$VATE DD-CKD

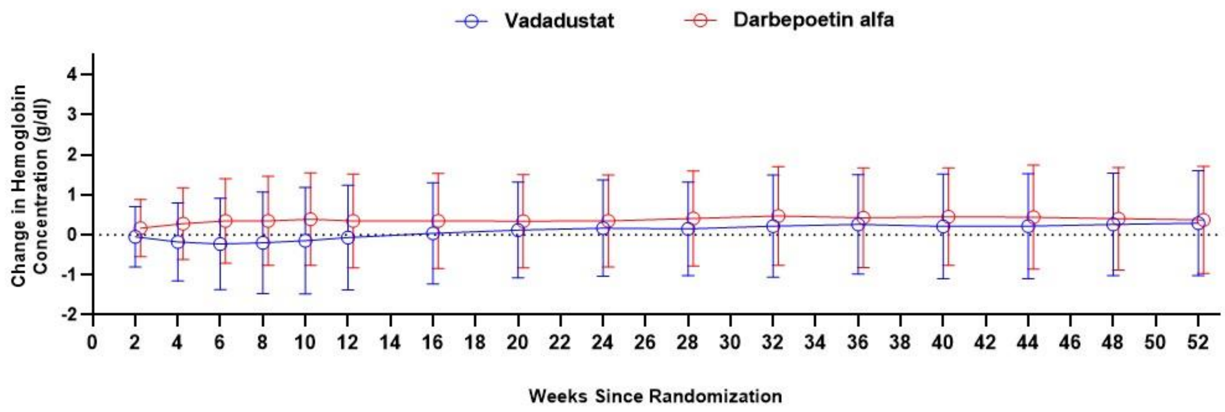

No. at Risk

Vadadustat	1021	980	970	972	950	966	961	938	907	889	875	861	835	810	782	797
Darbepoetin alfa	1016	1019	1007	993	967	983	990	979	949	918	913	895	882	868	849	850

C. ESA-Untreated - US

PRO$_2$TECT NDD-CKD

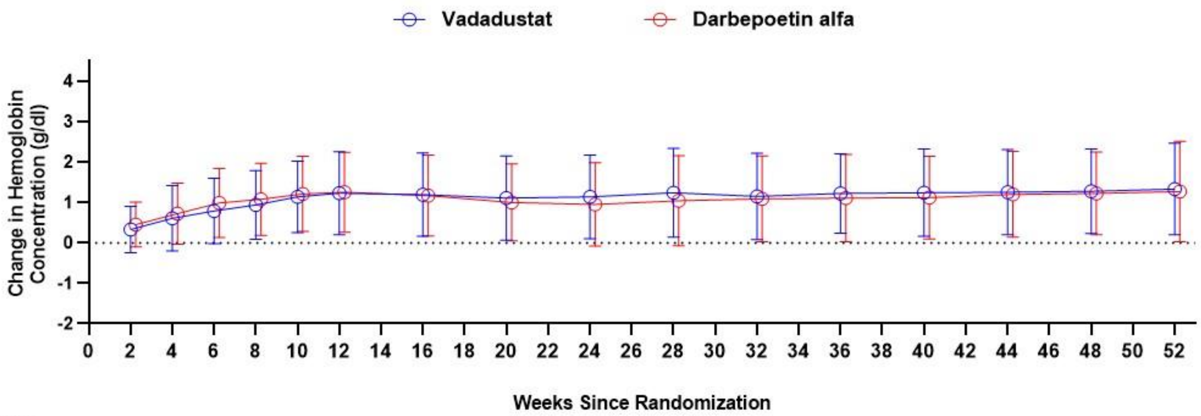

No. at Risk

Vadadustat	496	488	472	471	451	468	455	442	433	411	406	393	372	351	342	338
Darbepoetin alfa	490	483	461	454	445	459	453	437	429	411	408	390	368	346	339	323

D. ESA-Treated - US **PRO_2TECT NDD-CKD**

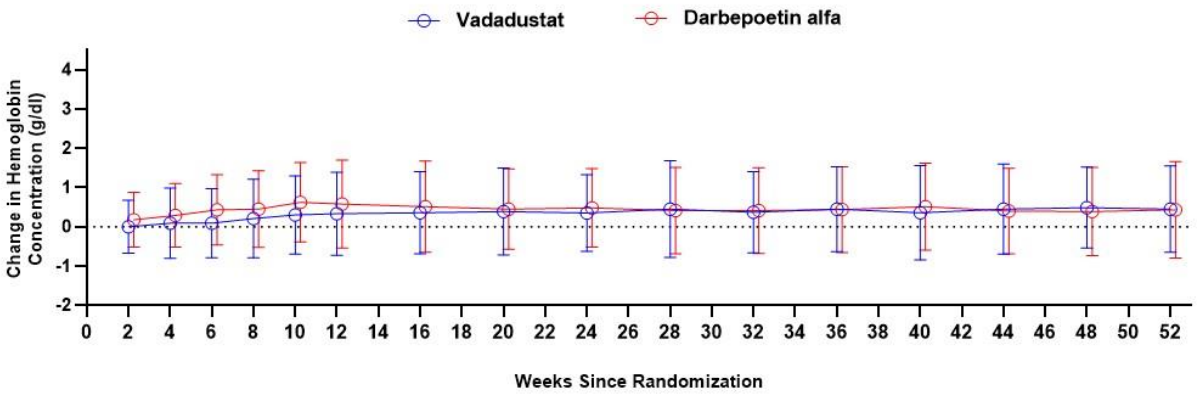

No. at Risk

Vadadustat	310	300	289	288	290	290	285	282	269	262	267	253	236	229	214	211
Darbepoetin alfa	311	316	306	306	296	304	298	290	296	282	279	267	244	230	221	217

E. Incident Dialysis – Non-US

INNO$_2$VATE DD-CKD

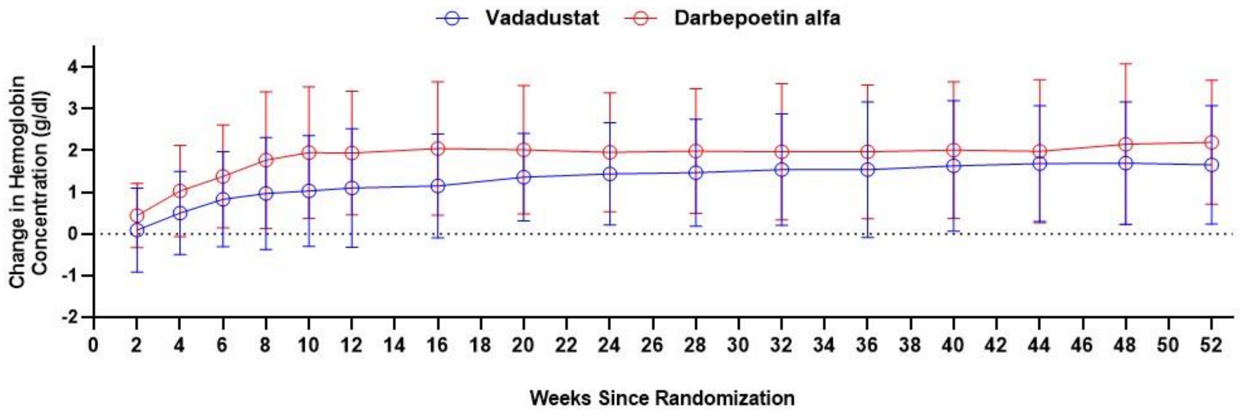

No. at Risk

Vadadustat	76	76	74	75	70	76	75	70	70	66	65	69	60	55	46	45
Darbepoetin alfa	83	80	83	79	81	82	80	82	78	78	79	75	68	59	54	50

F. Prevalent Dialysis – Non-US INNO$_2$VATE DD-CKD

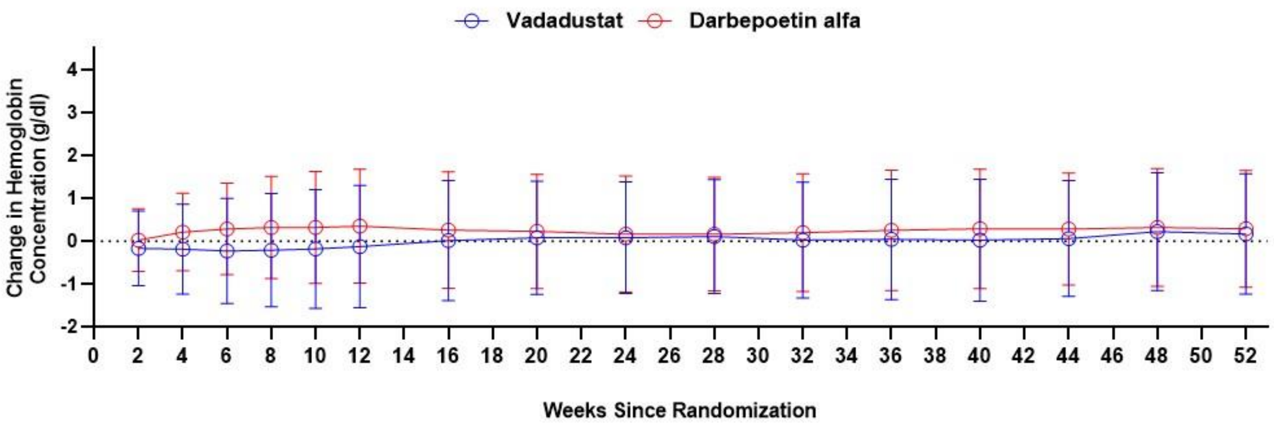

No. at Risk

Vadadustat	642	645	639	618	632	619	629	610	603	594	582	573	556	515	471	435
Darbepoetin alfa	642	649	647	626	636	622	635	625	608	596	598	590	574	527	474	444

G. ESA-Untreated – Non-US

PRO_2TECT NDD-CKD

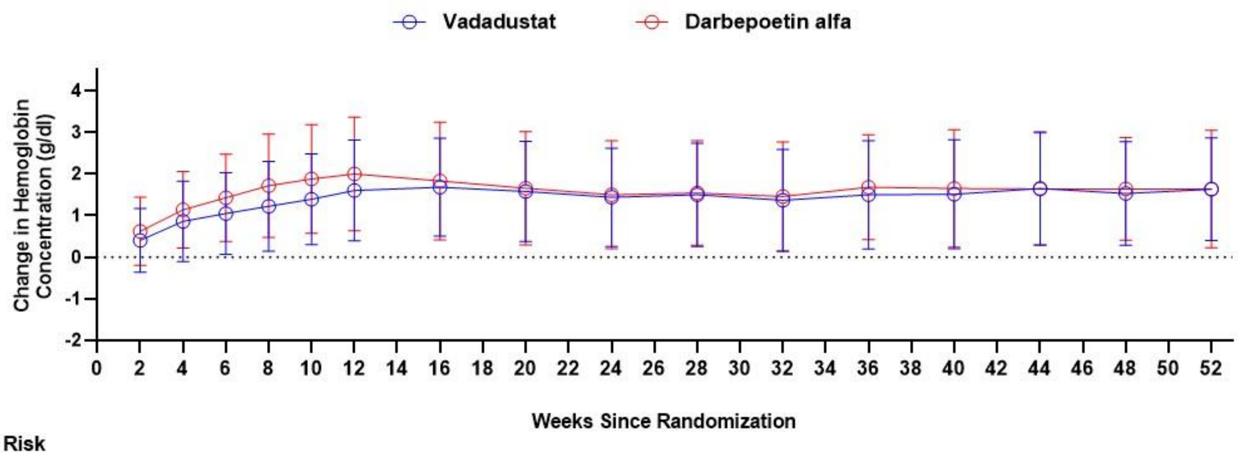

No. at Risk

Vadadustat	325	316	304	308	291	302	295	292	279	265	269	248	222	204	201	192
Darbepoetin alfa	321	323	317	318	313	310	313	306	302	287	282	281	246	237	220	220

H. ESA-Treated – Non-US

PRO$_2$TECT NDD-CKD

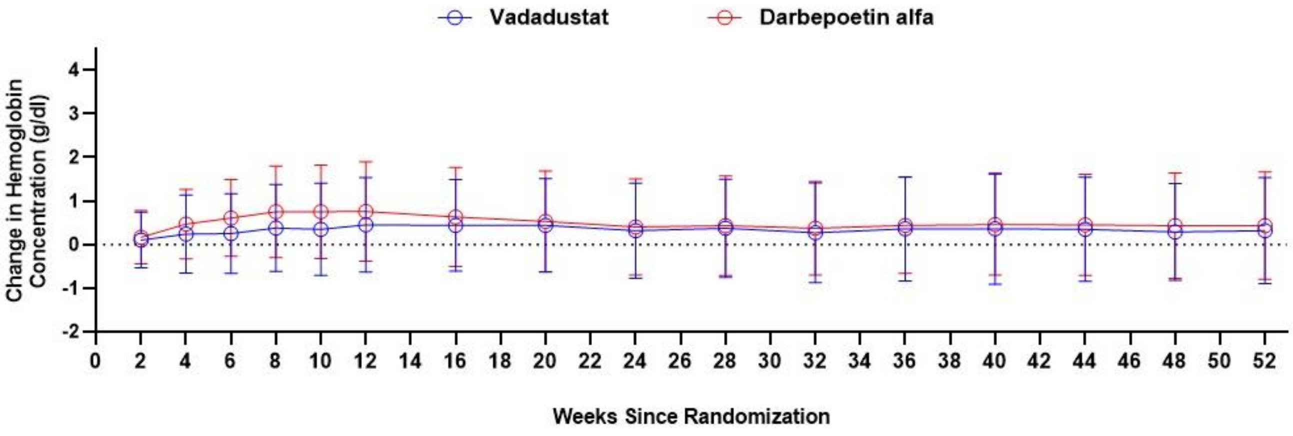

No. at Risk																
Vadadustat	501	501	502	493	488	483	486	479	475	456	445	421	381	357	331	331
Darbepoetin alfa	500	491	486	493	485	490	488	483	471	469	465	446	393	374	352	342

CKD, chronic kidney disease; DD-CKD, dialysis-dependent CKD; ESA, erythropoiesis-stimulating agent; NDD-CKD, non-dialysis dependent CKD.
